# Feasibility of Single-Port Access (SPA) Laparoscopy for Large Ovarian Tumor Suspected to Be Borderline Ovarian Tumor

**DOI:** 10.3389/fonc.2020.583515

**Published:** 2020-09-16

**Authors:** Jun-Hyeok Kang, Joseph J. Noh, Soo Young Jeong, Jung In Shim, Yoo-Young Lee, Chel Hun Choi, Jeong-Won Lee, Byoung-Gie Kim, Duk-Soo Bae, Hyun-Soo Kim, Tae-Joong Kim

**Affiliations:** ^1^Division of Gynecologic Oncology, Department of Obstetrics and Gynecology, Samsung Medical Center, Sungkyunkwan University School of Medicine, Seoul, South Korea; ^2^Department of Pathology and Translation Genomics, Samsung Medical Center, Sungkyunkwan University School of Medicine, Seoul, South Korea

**Keywords:** borderline ovarian tumor, single-port access, laparoendoscopic single-site, laparotomy, large ovarian tumor

## Abstract

**Objectives:**

To compare the surgical, pathological and oncological outcomes of single-port access (SPA) laparoscopy against laparotomy for large ovarian tumor (>15 cm) suspected to be a borderline ovarian tumor (BOT) on preoperative imaging.

**Methods:**

A retrospective review of the patients who underwent SPA laparoscopy (SPA Group) or laparotomy (Laparotomy Group) for suspected BOT was performed. Surgical outcomes, including the rates of iatrogenic spillage of tumor contents, and oncological outcomes, such as recurrence-free survival (RFS) and overall survival (OS), were compared between the two groups. Correlation between intraoperative frozen section analysis and permanent pathology results was also assessed.

**Results:**

A total of 178 patients underwent surgical treatment for suspected large BOT. Among them, 105 patients with a mean tumor diameter of 20.9 ± 6.5 cm underwent SPA laparoscopy, and the other 73 patients, with a mean tumor diameter 20.2 ± 5.9 cm, underwent laparotomy. The mean operation time did not differ between the two groups (99.1 ± 41.9 min for SPA Group *vs*. 107.3 ± 35.7 min for Laparotomy Group, *p* = 0.085). There was no difference in the occurrence of iatrogenic spillage of tumor contents between the groups either (11.4% in the SPA Group *vs*. 6.8% in the Laparotomy Group, *p* = 0.381). However, the postoperative complication rates were significantly higher in the Laparotomy Group compared with SPA Group (16.4% *vs*. 5.7%, *p* = 0.025). The surgical approach was not associated with the misdiagnosis rates of frozen section analysis (19% in the SPA Group *vs*. 26% in the Laparotomy Group, *p* = 0.484). The most common histologic type of the tumors was mucinous in both groups.

**Conclusion:**

SPA laparoscopy is feasible, safe, and not inferior to laparotomy for surgical treatment of large ovarian tumors that suspected to be BOT on preoperative imaging.

## Introduction

A borderline ovarian tumor (BOT), characterized as a tumor with a low potential for malignancy, accounts for approximately 10 to 15% of all epithelial ovarian tumors (1–3). It has distinguishing clinical features that are intermediate between benign ovarian tumors and invasive ovarian cancer. Although BOT can manifest as a metastatic disease and can recur, similar to malignant ovarian cancer, it generally behaves benignly and is usually diagnosed at an early stage and a younger age. For these reasons, it has excellent prognosis compared to malignant ovarian cancer (4). The current standard treatment for BOT is complete staging, including hysterectomy, bilateral salpingo-oophorectomy (BSO), omentectomy, cytology analysis, and resection of all suspicious tissues, similar to surgery for malignant tumors (5). However, conservative surgery is acceptable in premenopausal women who want to preserve fertility due to its favorable prognosis.

With an increasing demand for minimally invasive surgery (MIS) in the gynecologic field, single-port access (SPA) laparoscopy has been widely implemented owing to its benefits over conventional laparoscopy or laparotomy (6). However, in case of large ovarian tumors suspected to be BOT on imaging evaluations, concerns remain among surgeons with regard to the adequacy of SPA laparoscopy as a surgical approach because of its difficulties in surgical techniques, fear of intraperitoneal spillage of tumor contents, and lack of data on oncological outcomes. Furthermore, the accuracy and reliability of frozen-section (FS) analysis for BOT are less established, especially for tumors larger than 15 cm along its longest dimension. Previous studies have suggested the feasibility of SPA laparoscopy for BOT, but most of these studies were conducted on patients with a relatively small BOT that could be excised with ease (7, 8). For these reasons, surgeons are reluctant to perform SPA laparoscopy for BOT with a large tumor size.

In the present study, we performed a retrospective review of patients with large ovarian tumors (greater than 15 cm along the longest dimension when viewed by imaging) suspected to be BOT and who thus underwent surgical treatment. The primary aim of the study was to evaluate the surgical and oncological outcomes of the patients who underwent SPA laparoscopy in comparison to those who underwent laparotomy. The secondary aim was to review the correlation between the intraoperative FS analysis and the final histopathological diagnosis.

## Materials and Methods

### Patient Selection

A retrospective review of the patients who underwent either SPA laparoscopy (SPA Group) or laparotomy (Laparotomy Group) for suspected BOT was performed. The patients who underwent surgical treatment between January 2015 and December 2018 at an urban tertiary academic medical center (Samsung Medical Center) in Seoul, South Korea, were included. The patients who met the following criteria were eligible for the study: (1) those with an ovarian tumor suspected to be BOT and that measured greater than 15 cm along its longest dimension, when viewed on preoperative imaging modalities such as computed tomography (CT) or magnetic resonance imaging (MRI); (2) those who underwent SPA laparoscopy or laparotomy for surgical removal of the tumor; and (3) those who had intraoperative FS analysis. The patients who were excluded were: (1) those who did not have a pathological report of FS during surgery, (2) those whose preoperative imaging evaluations were suggestive of malignancy as the most likely diagnosis, and (3) those who had had a previous pathological diagnosis of BOT or ovarian cancer before the present surgery. The study was approved by the Institutional Review Board of Samsung Medical Center.

### Data

The data collected was age, parity, body mass index (BMI), tumor size, preoperative serum tumor marker levels (CA-125 and CA 19–9), and history of prior abdominal surgery. The type of initial surgical approach (either SPA laparoscopy or laparotomy), the extent of surgical procedures performed, total operation time, the occurrence of intraperitoneal spillage of tumor contents, estimated blood loss (EBL), perioperative complications, duration of hospital stay, and conversion rates to multiport laparoscopy or laparotomy from SPA laparoscopy were also assessed. We also evaluated the diagnostic accuracy and reliability of FS analysis. Radical surgery was defined as BSO with or without hysterectomy, while conservative surgery was defined as the preservation of at least part of one ovary and the uterus. Complete staging consisted of resection of all suspicious tumor tissues, peritoneal washing cytology, multiple random peritoneal biopsies, infracolic omentectomy, BSO, and hysterectomy. An obvious gross rupture of the cystic wall during surgery was considered as intraperitoneal spillage. Hospital stay was defined as the number of days between the operation date and discharge. Total operative time was defined as the time from the initial incision to the completion of wound closure. All FS analyses were performed by a single board-certified pathologist specialized in gynecologic oncology. At least two representative sections were obtained for each patient.

### Surgical Techniques of SPA Laparoscopy

The type of initial surgical approach was decided comprehensively considering the characteristics of the ovarian tumor, the severity of the predicted intra-abdominal adhesion, the surgeon’s preference, and the patient’s demand. The extent of surgical procedures (radicality of surgery) depended on the results of the intraoperative FS analysis, age of the patient, and patient’s desire to preserve fertility. The surgical techniques of SPA laparoscopy have been described previously (9). A small vertical transumbilical incision of 2.0 to 2.5 cm was made using the open Hasson technique while taking care not to rupture the ovarian tumor. A single-port wound retractor (Glove Port^TM^, Nelis, Inc., Seoul, South Korea; Supplementary Figure 1A) was inserted through the umbilicus to expose the surface of the ovarian tumor through umbilical incision, and the inner edge of the wound retractor was covered with a surgical gauze to minimize the leakage of cystic contents during puncture and aspiration. Two 2–0 absorbable traction sutures or Kelly clamp traction was applied at both opposite ends of the cystic surface exposed through the wound retractor. While lifting the cystic wall with traction sutures or Kelly clamps, the cystic fluid was aspirated using a suction system after the puncture of the cyst with an OCHSNER trocar (Cardinal Health, Inc., Dublin, OH, United States; Supplementary Figures 1B–D). Once the fluid contents were aspirated to the maximum extent possible, the OCHSNER trocar was removed, and the puncture site was sutured (Supplementary Figure 1E). The tumor was then rolled intraperitoneally to find another potential puncture site, because tumors often have multiple septate sections. Additional puncture and drainage was performed in the same manner. When the size of the tumor was reduced enough for the laparoscopic approach, a multichannel cap was placed on the wound retractor. Pneumoperitoneum with carbon dioxide at 12 mmHg was established. First, peritoneal washing cytology was performed. SPA laparoscopy was performed with a rigid 30-degree, 5-mm laparoscope, conventional rigid straight laparoscopic instruments, and 5-mm articulating laparoscopic grasper (Roticulator^TM^, Covidien, Inc., Mansfield, MA, United States). After the resection of ovarian tumor, the resected ovarian tumor was wrapped with an endo-pouch, and cold knife morcellation through the umbilical opening was performed with the tumor still inside the bag (Supplementary Figure 1F). The whole ovarian tumor tissues were sent for FS analysis. Once FS analysis revealed BOT or malignancy, laparoscopic or laparotomic surgical staging was continued.

### Statistical Analyses

All statistical analyses were performed using SPSS version 25.0 (SPSS, Inc., Chicago, IL, United States). The normality of the data was assessed using the Shapiro–Wilk test. Statistical significance was determined using the Fisher’s exact test for dichotomous variables and using the independent Student’s *t*-test for continuous variables. Survival curves were calculated according to the Kaplan–Meier method with the log-rank test. *p*-values less than 0.05 were considered significant.

## Results

**FIGURE 1:**
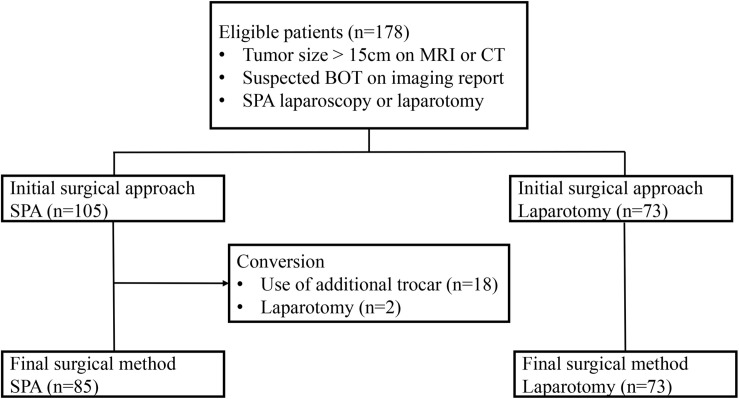
Flow chart for the studied patients.

During the study period, a total of 178 patients were included in the study (Figure 1). Among them, 105 (58.9%) underwent SPA laparoscopy, while 73 (41.1%) were treated with laparotomy. The baseline characteristics are summarized in Table 1. The mean size of the ovarian tumors in all patients (*n* = 178) was 20.6 ± 6.3 cm, and there was no significant difference between the two groups in this regard (20.9 ± 6.3 cm for the SPA Group *vs*. 20.2 ± 5.9 cm for the Laparotomy Group, *p* = 0.494). The patients in the SPA Group (39.8 ± 15.9 years) were significantly younger than those in the laparotomy group (46.2 ± 15.5 years, *p* = 0.009). The levels of CA 19–9 were significantly lower in the SPA Group compared to the Laparotomy Group [5.3 (2.9–13.4) IU/mL for the SPA Group *vs*. 9.8 (4.3–33.8) IU/mL for the Laparotomy Group, *p* = 0.029]. In the Laparotomy Group, the majority of the patients were postmenopausal and had histories of one or more previous abdominal surgeries. However, there were no significant differences in parity, preoperative symptoms, CA-125 levels, and BMI between the two groups. Twenty (19.0%) patients in the SPA Group experienced conversion to laparotomy (*n* = 2) or required the use of additional trocars (*n* = 18). However, all conversions were done after removal of the tumor from the abdominal cavity using the SPA.

**TABLE 1:** Baseline characteristics of the patients.

Characteristics	Total	SPA laparoscopy	Laparotomy	*p*-value
	(*n* = 178)	(*n* = 105)	(*n* = 73)	
Age (years)		39.8 ± 15.9	46.2 ± 15.5	0.009*
**Parity**				
Nulliparous	80 (44.9)	53 (50.5)	27 (37.0)	0.075
Parous	98 (55.1)	52 (49.5)	46 (63.0)	
Menopause				
No	121 (67.9)	80 (76.2)	41 (56.2)	0.005*
Yes	57 (32.1)	25 (23.8)	32 (43.8)	
**Symptoms and sign**				
Incidental mass	103 (57.8)	61 (58.1)	42 (57.5)	0.242
Abdominal pain	37 (20.7)	26 (24.8)	11 (15.1)	
Abdominal distension	20 (11.2)	10 (9.5)	10 (13.7)	
Abdominal palpable mass	18 (10.3)	8 (7.6)	10 (13.7)	
Tumor size (cm)	20.6 ± 6.3	20.9 ± 6.5	20.2 ± 5.9	0.494
**Tumor marker**				
CA-125 (IU/mL)	11.9 (6.6–25.1)	11.3 (7.4–22.9)	13.5 (5.9–28.3)	0.133
CA19–9 (IU/mL)	7.4 (3.4–19.1)	5.3 (2.9–13.4)	9.8 (4.3–33.8)	0.029*
BMI (kg/m^2^)	23.4 ± 3.9	23.2 ± 3.5	23.8 ± 4.6	0.357
**Previous abdominal Op. Hx.**				
No	120 (67.4)	79 (75.2)	41 (56.2)	0.008*
Yes	58 (32.6)	26 (24.8)	32 (43.8)	
**Change in surgical approach**				
Use of additional port		18 (17.1)	N/A	
Conversion to laparotomy		2 (1.9)	N/A	

Values are presented as median (IQR) or n (%). SPA, single port access; Op., operation; Hx., history; BMI, body mass index; and N/A, not available. *p < 0.05.

The surgical outcomes, according to the initial surgical approach, are shown in Table 2. Rates of conservative surgery and incomplete staging were similar between the two groups. The mean operative time did not differ between the two groups. However, the length of postoperative hospital stay was shorter in the SPA Group than in the Laparotomy Group [2 (2–3) days *vs*. 5 (4–5) days, *p* = 0.041]. The incidence of perioperative complications, including ileus and wound complications, was also lower in the SPA Group than in the Laparotomy Group [6 complications (5.7%) *vs*. 15 complications (20.5%), *p* = 0.025]. Other surgical outcome parameters such as the occurrence of intraperitoneal spillage of tumor contents, EBL, and changes in hemoglobin levels before and after the operations did not demonstrate any differences between the two groups.

**TABLE 2:** Surgical outcomes according to the initial surgical approach.

	SPA laparoscopy	Laparotomy	*p*-value
	(*n* = 105)	(*n* = 73)	
Radicality of surgery			0.117
Conservative surgery	79 (75.2)	47 (64.4)	
Radical surgery	26 (24.8)	26 (35.6)	
Completeness of surgery			0.141
Incomplete staging	69 (65.7)	40 (54.8)	
Complete staging	36 (34.3)	33 (45.2)	
Operation time (min)			
Mean ± SD	99.1 ± 41.9	107.3 ± 35.7	0.085
Estimated blood loss (mL)			
Median (range)	80 (50–100)	100 (50–150)	0.051
Spillage of cystic content			0.307
No	93 (88.6)	68 (93.2)	
Yes	12 (11.4)	5 (6.8)	
Change of Hb (g/dL)			
Mean ± SD	1.7 ± 0.9	1.6 ± 0.8	0.408
Hospital stay (days)			
Median (range)	2 (2–3)	5 (4–5)	0.041*
Intraoperative complications			0.631
Bowel injury	1	2	
Bladder injury	0	0	
Ureter injury	0	0	
Vessel injury	1	1	
Postoperative complications			0.025*
Ileus	1	4	
Vaginal cuff dehiscence	1	0	
Wound dehiscence or infection	2	8	

Values are presented as n (%). SPA, single port access. *p = 0.05.

The final pathological analysis revealed that 99 of 178 patients (55.6%) had a benign ovarian tumor, 54 patients (30.4%) had BOT, and 25 patients (14.0%) had a malignant tumor (Supplementary Table S1). The most common histological subtype was mucinous tumor (71.9%) followed by serous tumor (13.5%). The rates of benign tumor, BOT, and malignant tumor were similar between the two groups. Agreement between the FS analysis and final pathology was observed in 139 of 178 patients (78.0%), yielding an overall sensitivity and positive predictive value (PPV) of 66.8% and 80.6%, respectively (Table 3 and Supplementary Table S2). Over-diagnosis of FS analysis was identified in 9 out of 178 patients (5%). In such patients, the FS analysis revealed BOT while the final pathology revealed a benign tumor, or the FS analysis revealed malignancy while the final pathology revealed BOT or benign tumor. Under-diagnosis of FS analysis was more frequently observed (30 of 178 patients, 16.8%). In those patients, the FS analysis revealed BOT or benign tumor while the final pathology revealed malignancy, or the FS analysis revealed a benign tumor while the final pathology revealed BOT or malignancy.

**TABLE 3:** Comparison of misdiagnosis and same-diagnosis of frozen sections according to the surgical approach.

	Frozen section diagnosis compared with permanent diagnosis				
Frozen section	Total	Under-diagnosis	Same-diagnosis	Over-diagnosis	*p-*value
Total (*n* = 178)					0.484
SPA	105	16 (15.2)	85 (81.0)	4 (3.8)	
Laparotomy	73	14 (19.2)	54 (74.0)	5 (6.8)	
Benign (*n* = 113)					0.426
SPA	70	12 (17.1)	58 (82.9)	N/A	
Laparotomy	43	10 (19.5)	33 (80.5)	N/A	
BOT (*n* = 52)					0.642
SPA	31	4 (12.9)	23 (74.2)	4 (12.9)	
Laparotomy	21	4 (15.4)	13 (69.2)	4 (15.4)	
Malignancy (*n* = 13)					1.000
SPA	4	N/A	4 (100)	0	
Laparotomy	9	N/A	8 (88.9)	1 (11.1)	

*Values are presented as n (%). SPA, single port access; BOT, borderline ovarian tumor; and N/A, not available.*

**FIGURE 2:**
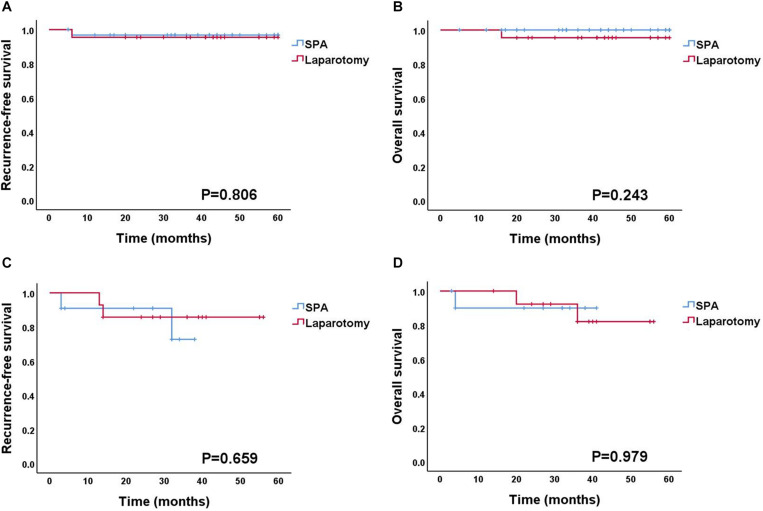
Survival outcomes according to the surgical approach. **(A,B)** Recurrence-free survival and overall survival for BOT patients. **(C,D)** Recurrence-free survival and overall survival for OC patients. BOT, borderline ovarian tumor; OC, ovarian cancer.

The details of surgical findings are presented in Table 4. The majority of the patients with BOT were International Federation of Gynecology and Obstetrics (FIGO) stage IA (88.8%). Among the patients with malignant tumors, FIGO stage I and grade I were the most common (88% and 68%, respectively). The median follow-up duration of patients with BOT or malignant tumor was 39 months (range: 27–48 months). Two BOT patients experienced recurrence (one patient in the SPA Group and one in the Laparotomy Group), and the patient in the Laparotomy Group died of the disease at 16 months. Among the patients with a malignant tumor, four patients experienced recurrence (two in the SPA Group and two in the Laparotomy Group), and three patients died of the disease (one in the SPA Group and two in the Laparotomy Group). The recurrence-free survival (RFS) and overall survival (OS) of the patients diagnosed with either BOT or malignant tumor did not differ between the two surgical approaches (Figure 2).

**TABLE 4:** Tumor characteristics of the patients diagnosed with above borderline malignancy and concordance between the two pathological findings.

	Final diagnosis OC	Final diagnosis BOT
	(*n* = 25)	(*n* = 54)
Stage		
IA	12 (48.0)	48 (88.8)
IB	–	2 (3.7)
IC	10 (40.0)	4 (7.5)
II	–	–
III	3 (12.0)	–
Grade		
Grade 1	17 (68.0)	–
Grade 2	2 (8.0)	–
Grade 3	6 (24.0)	–
Final surgical method		
SPA	7 (28.0)	26 (48.1)
SPA with additional port	2 (8.0)	6 (11.1)
Laparotomy	16 (64.0)	22 (40.8)
Frozen section finding		
Benign	5	17
Borderline	8	36
Malignancy	12	1

*Values are presented as n (%). SPA, single port access; OC, ovarian cancer; and BOT, borderline ovarian tumors.*

## Discussion

The results of the present study demonstrated the feasibility of SPA laparoscopy for surgical treatment of a large ovarian tumor suspected to be BOT on imaging evaluations. Compared with laparotomy, SPA laparoscopy was associated with a shorter hospital stay and less perioperative complications. Furthermore, there were no differences in pathological and oncological outcomes between the patients who received SPA laparoscopy and those who received laparotomy.

Despite the benefits of SPA laparoscopy illustrated in previous studies, many surgeons remain reluctant to perform a single-port laparoscopic approach for large BOT due to the aforementioned reasons. However, since the introduction of SPA laparoscopy, the advantages of SPA with an extracorporeal approach for large ovarian tumors have been reported in several studies (10–12). It not only provides better cosmesis, less pain, and faster recovery after surgery but the relatively large umbilical incision created in SPA also facilitates extracorporeal procedures and the removal of bulky specimens with ease.

Large tumor volumes of BOT are oftentimes the major obstacle to the laparoscopic approach for surgeons. The mean tumor size of the patients who underwent SPA laparoscopy in the present study was over 20 cm, which did not differ from the patients who underwent laparotomy. Previous studies have also demonstrated the feasibility of SPA laparoscopy for large ovarian tumors. In those studies, surgeons were able to remove tumors with a mean size of 20 cm with SPA laparoscopy (10, 12). Almost all patients underwent successful surgery without conversion to laparotomy. Therefore, tumor size should not deter surgeons from performing SPA laparoscopy for surgical removal of BOT.

Another concern regarding the SPA laparoscopic approach for large BOT is the possibility of intraperitoneal spillage of tumor contents. Surgeons often feel at ease performing manipulations with a multiport approach because of the expectation that multiport approach would reduce the possibility of tumor spillage. However, SPA laparoscopy may provide advantages over the conventional laparoscopic approach with respect to the spillage of tumor contents. First, using the open Hasson technique and umbilical wound retractor, surgeons can safely expose ovarian tumors through the umbilicus without causing a cystic wall injury, which frequently occurs during blind trocar insertions in the conventional laparoscopic approach. Second, this approach can provide a clear surgical view of the abdominal cavity without the need for establishing pneumoperitoneum. Furthermore, volume reduction by extracorporeal aspiration of cystic contents can reduce the occurrence of rupture during intracorporeal manipulation. According to propensity score matching analysis for large ovarian tumors, conventional laparoscopy had the advantages of shorter operation time and less intraoperative bleeding compared with laparotomy, but it had a higher spillage rate than laparotomy (54.5% vs. 12.1%, *p* < 0.001) (13). On the other hand, one study demonstrated that the spillage rates of tumor contents during ovarian cystectomy were significantly lower in SPA laparoscopy compared to conventional laparoscopy (11). In the present study, 17 patients experienced intraperitoneal spillage of tumor contents. However, there was no difference in spillage rates between the patients who underwent SPA laparoscopy and those who underwent laparotomy. The spillage rates observed in the present study (11.4%) are similar to that previously reported (8–10%) (10). The effects of intraperitoneal spillage of tumor contents of BOT on prognosis is still unknown (14–19). According to a retrospective study in which the authors evaluated the prognostic factors for early stage ovarian cancer in 1,545 patients, rupture during surgery was found to be associated with shorter disease-free survival (HR 1.64, *p* = 0.022) (14). In contrast, a meta-analysis showed that intraperitoneal rupture of early stage ovarian cancer did not increase the risk of disease recurrence in patients who received complete staging along with adjuvant platinum-based chemotherapy (16). For BOT patients, the association of intraperitoneal spillage of tumor contents with the risk of disease recurrence is not clearly understood yet (20, 21). Although definitive evidence is still lacking that demonstrates that intraperitoneal spillage of tumor contents may increase the risks of disease recurrence in BOT, meticulous efforts should be undertaken to prevent the rupture of tumor contents.

The potential misdiagnosis of FS analysis is also a concern in SPA laparoscopy for large BOT. Establishing an accurate preoperative diagnosis of BOT is difficult due to its intermediate features between benign and malignancy. Even MRI imaging, which is known to have high diagnostic accuracy for BOT, has been reported to have only 45.5% sensitivity and 96.1% specificity (22, 23). In particular, it is known that the differential diagnosis of seromucinous BOT and atypical endometriosis, associated with endometriosis-associated ovarian cancer, on preoperative imaging is challenging. Therefore, most gynecologic oncologists have no choice but to depend on intraoperative FS analysis when they decide on the extent of surgical procedures. Therefore, achieving high accuracy and reliability of FS analysis is essential because the misdiagnosis of FS analysis could result in undertreatment or overtreatment. In the present study, the overall agreement between FS analysis and the final pathologic report was 78%. The sensitivity and PPV of FS analysis for BOT were 66.6% and 69.2%, respectively. These values are relatively lower than has been previously reported (sensitivity between 75 and 93% and PPV between 88 and 95%) (24, 25). Underdiagnosis was identified in 30 patients (16.8%) in the present study, which was relatively higher than that reported in previous studies (24). Such discrepancies in misdiagnosis could be explained by the relatively large tumor size and the fact that the most common histological subtype of the tumor in the present study was a mucinous tumor, which often contains benign, borderline, and malignant components together (24, 26, 27). However, the rates of misdiagnosis in the FS analysis did not differ between the SPA Group and the Laparotomy Group in the present study. Similar findings have also been reported in previous studies. According to a meta-analysis that evaluated the misdiagnosis rates of FS analysis in BOT, the surgical approach (laparoscopy *vs*. laparotomy) was not associated with the accuracy of FS analysis (OR = 1.34, CI 0.57–3.11, *p* = 0.50) (24).

The oncological outcomes of SPA laparoscopy for BOT in comparison to laparotomy were also comparable, according to the results of the present study. Similar survival rates of patients with BOT who underwent different surgical approaches have been reported by other studies as well (7, 20). Moreover, these similar oncological outcomes among various surgical approaches were also demonstrated in patients with early stage ovarian cancer. Based on these results, the National Comprehensive Cancer Network (NCCN) guidelines have supported the use of MIS as a potential surgical approach for selected patients with early stage ovarian cancer. Robotic assisted laparoscopy (RAL), which is the most advanced technology for MIS, can be considered as a possible treatment alternative for BOT and early stage ovarian cancer (28). However, there are some considerations for applying RAL to a huge ovarian tumor, similar to that seen in our study cohort. Robotic surgery has clear technical advantages over SPA laparoscopy. Limitations in space and movement of SPA laparoscopy lead to conflicts among instruments, thus inhibiting careful manipulation. Therefore, SPA laparoscopy usually requires a long learning curve, and skilled surgeons most often undertake the procedure (29, 30). On the other hand, a relatively large umbilical hole created in SPA, compared with RAL, facilitates further extracorporeal procedures and aids in the removal of the bulky specimen. This limitation of conventional RAL for huge ovarian tumors can be overcome if a single-port platform is used instead of an umbilical 8–12 mm trocar [e.g., conventional RAL with a single-port platform, single-site robot surgery, and single-port robot surgery (da Vinci^®^ SP Surgical System)]. Of course, these types of robotic surgery for huge ovarian tumors may take a longer time to perform because undocking and redocking is required for manual extracorporeal procedures (hybrid technique). However, it can be an attractive surgical method that combines the precision of RAL with the extracorporeal procedure advantages of SPA. To the best of our knowledge, there is no study evaluating the feasibility of RAL for BOT. This will be an interesting subject for the future research.

The strength of this study is that, to date, it is the first study to compare SPA laparoscopy and laparotomy only for huge ovarian tumor suspected as BOT. However, there were some limitations that need to be mentioned. First, this was a retrospective study. Second, there was no pathological review conducted, which could have resulted in inaccurate diagnoses in some patients. Third, there was no evaluation in terms of postoperative pain and cosmetic outcomes, which are some of the other advantages of SPA over laparotomy. Fourth, SPA laparoscopy for huge ovarian tumors suspected to be BOT is not a general surgical method but is a technique mainly performed in a tertiary medical center by an experienced surgeon. Fifth, the spillage was assessed by a relatively subjective parameter of gross cystic wall rupture, not by cytologic confirmation though washing cytology before and after the removal of the ovarian tumor.

In conclusion, SPA laparoscopy is a feasible, safe technique that is not inferior to laparotomy in terms of surgical, pathological, and oncological outcomes when managing large ovarian tumors suspected to be BOT. We recommend that physicians consider SPA laparoscopy, regardless of tumor size, as an initial approach, instead of laparotomy, which has a higher morbidity rate.

## Data Availability Statement

The datasets generated for this study are available on request to the corresponding author.

## Ethics Statement

The studies involving human participants were reviewed and approved by Institutional Reveiew Board of Samsung Medical Center. Written informed consent for participation was not required for this study in accordance with the national legislation and the institutional requirements.

## Author Contributions

J-HK and T-JK conceived and designed the study. J-HK, H-SK, B-GK, D-SB, and T-JK collected the data. J-HK, JJN, SYJ, JIS, Y-YL, CHC, J-WL, and T-JK performed data analysis and interpretation. J-HK, JJN, and T-JK contributed to writing and review and editing. All authors contributed to the article and approved the submitted version.

## Conflict of Interest

The authors declare that the research was conducted in the absence of any commercial or financial relationships that could be construed as a potential conflict of interest.
