# Supplementary material for: Feasibility of Single-Port Access (SPA) Laparoscopy for Large Ovarian Tumor Suspected to Be Borderline Ovarian Tumor
Source: Front Oncol. 2020 Sep 16;10:583515. doi: 10.3389/fonc.2020.583515 (PMC7526335; doi:10.3389/fonc.2020.583515)
Supplement: Supplementary file 1 [file Data_Sheet_1.docx]

**Supplementary Table S1 |** Diagnosis based on frozen sections compared with diagnosis based on permanent pathology.

|  | **Permanent** | | |  |
| --- | --- | --- | --- | --- |
| **Frozen** | **Benign** | **Borderline** | **Malignant** | **Total** |
| **All patients (*n* = 178)** |  |  |  |  |
| Benign | 91 | 17 | 5 | 113 |
| Borderline | 8 | 36 | 8 | 52 |
| Malignant | 0 | 1 | 12 | 13 |
| Total | 99 | 54 | 25 | 178 |
| **SPA group (*n* = 105)** |  |  |  |  |
| Benign | 58 | 9 | 3 | 70 |
| Borderline | 4 | 23 | 4 | 31 |
| Malignant | 0 | 0 | 4 | 4 |
| Total | 62 | 32 | 11 | 105 |
| **Laparotomy (*n* = 73)** |  |  |  |  |
| Benign | 33 | 8 | 2 | 43 |
| Borderline | 4 | 13 | 4 | 21 |
| Malignant | 0 | 1 | 8 | 9 |
| Total | 37 | 22 | 14 | 73 |

*Values are presented as n (%).*

*SPA, single port access.*

**Supplementary Table S2 |** Details of the final pathological diagnosis according to the initial surgical approach.

| **Final pathology** | **Total**  **(*n* = 178)** | **SPA laparoscopy**  **(*n* = 105)** | **Laparotomy**  **(*n* = 73)** | ***p*-value** |
| --- | --- | --- | --- | --- |
|  |  |  |  | 0.239 |
| **Benign** | 99 | 62 (100) | 37 (100) |  |
| Mucinous cystadenoma | 68 | 43 (69.4) | 25 (67.6) |  |
| Serous cystadenoma | 13 | 10 (16.1) | 3 (8.1) |  |
| Mature cystic teratoma | 8 | 5 (8.1) | 3 (8.1) |  |
| Endometrioma | 6 | 4 (6.4) | 2 (5.5) |  |
| Fibroma | 2 | 0 | 2 (5.5) |  |
| Thecoma | 1 | 0 | 1 (2.6) |  |
| Lipoma | 1 | 0 | 1 (2.6) |  |
| **Borderline malignancy** | 54 | 32 (100) | 22 (100) |  |
| Mucinous borderline tumor | 47 | 26 (81.2) | 20 (90.1) |  |
| Serous borderline tumor | 4 | 3 (9.4) | 2 (0.9) |  |
| Seromucinous borderline tumor | 3 | 3 (9.4) | 0 |  |
| **Malignancy** | 25 | 11(100) | 14 (100) |  |
| Mucinous carcinoma | 14 | 6 (54.5) | 8 (57.2) |  |
| Serous carcinoma | 6 | 3 (27.2) | 3 (21.4) |  |
| Clear cell carcinoma | 2 | 2 (18.3) | 0 |  |
| Immature teratoma | 3 | 0 | 3 (21.4) |  |

*Values are presented as n (%).*

*SPA, single port access.*
