# Supplementary figures and images for: Feasibility of Single-Port Access (SPA) Laparoscopy for Large Ovarian Tumor Suspected to Be Borderline Ovarian Tumor
Source: Front Oncol. 2020 Sep 16;10:583515. doi: 10.3389/fonc.2020.583515 (PMC7526335; doi:10.3389/fonc.2020.583515)

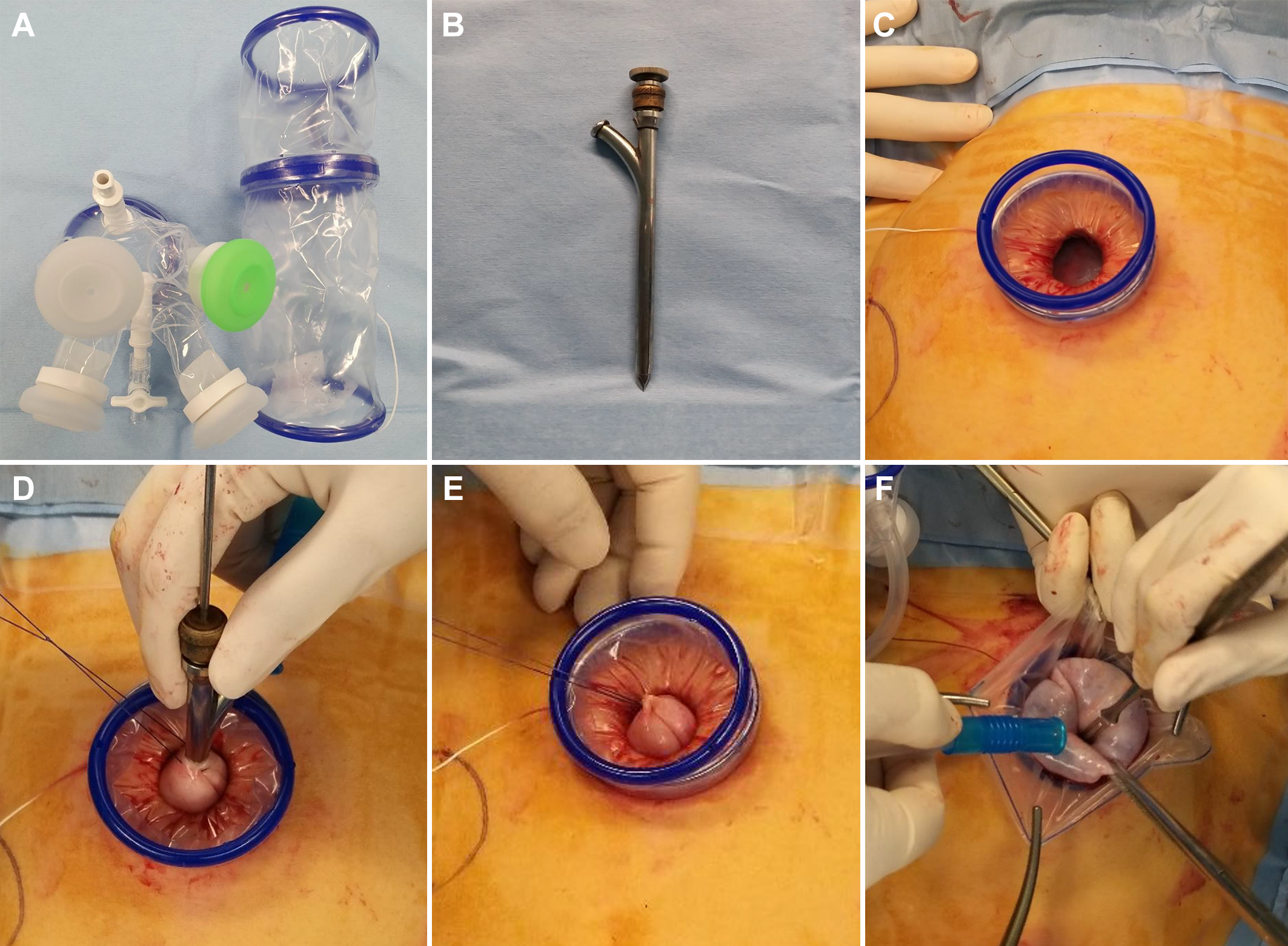

Supplement: Supplementary file 2 [file Image_1.TIF]
